# Factors Associated with Antibiotic Prescribing in Children with Otitis Media

**DOI:** 10.5402/2013/587452

**Published:** 2013-02-26

**Authors:** Christina T. Ryborg, Jens Søndergaard, Jørgen Lous, Anders Munck, Pia V. Larsen, Malene Plejdrup Hansen, Janus Laust Thomsen

**Affiliations:** ^1^Research Unit of General Practice, Institute of Public Health, University of Southern Denmark, J.B. Winsløws Vej 9A, 5000 Odense C, Denmark; ^2^Audit Project Odense, Research Unit of General Practice, Institute of Public Health, University of Southern Denmark, Denmark; ^3^Department of Clinical Microbiology, Odense University Hospital, Denmark

## Abstract

*Background*. Acute otitis media (AOM) is often treated with antibiotics. However, initial observation is recommended. Denmark has a low use of antibiotics compared with other countries, but the total use of antibiotics has increased by 32% during the last decade, and it is important to know whether general practitioners (GPs) prescribe antibiotics according to guidelines. *Objective*. The aim of the study was to analyse associations between GPs' antibiotic prescribing for AOM and symptoms, diagnoses, and characteristics of children. *Methods*. A cohort study where GPs consecutively included 954 children between 0 and 7 years old with a new ear symptom was carried out. The GPs registered symptoms, results of otoscopy and tympanometry, together with diagnosis and treatment. *Results*. Fever with and without earache was statistically associated with prescribing antibiotics, and it applies to both children up to two years of age (OR: 5.89 (confidence interval (CI): 2.62–13.21) and OR: 8.13 (CI: 4.61–14.32)) and children older than two years of age (OR: 4.59 (CI: 2.55–8.25) and OR: 19.45 (CI: 6.38–59.24)). A red tympanic membrane was statistically associated with the prescribing antibiotics in both age groups (0–2 years: OR: 4.73 (CI: 2.52–8.86) and >2–7 years: OR: 3.76 (CI: 2.13–6.64)). A flat tympanometry curve was only statistically associated with prescribing antibiotics in the oldest children (OR: 2.42 (CI: 1.17–5.00)). *Conclusion*. This study indicates that GPs to a large degree prescribe antibiotics appropriately according to guidelines.

## 1. Introduction

Acute otitis media (AOM) is a common infection during early childhood [1], often caused by bacteria. Antimicrobial agents have been the primary treatment for this infection since the 1950s. Antibiotic treatment is, however, associated with antimicrobial resistance which in many countries is increasing among bacterial pathogens causing AOM [2].

Denmark has a low use of antibiotics compared with other countries, and a study from 1998 found the Danish GPs' criteria for antibiotic treatment of AOM to be in accordance with recommendations, that is, primarily using nonantibiotic treatment and using penicillin V as the first choice of drug [3]. A recent study on prescription rates in six different countries (Lithuania, Kaliningrad, Spain, Argentina, Sweden, and Denmark) found that Denmark had a relatively low prescription rate of antibiotics for AOM compared to the other countries in the study (72.7%) [4]. However, the total use of antibiotics in Denmark has increased by 32% during the last decade (2001–2010) [5], and from 2009 to 2010 the total use of antibiotics in the primary sector in Denmark increased by 6%, reaching the highest level ever [5]. Little is known about the reasons for this increase, and it is important to know whether Danish GPs' criteria for antibiotic treatment of AOM are still in accordance with recommendations.

GPs normally use medical history and otoscopy when diagnosing children with ear symptoms, and a correct diagnosis is an important factor when assessing whether antibiotics should be prescribed to a child with ear symptoms. However, otoscopy has been shown to have unsatisfactory sensitivity and specificity regarding diagnosing of middle ear with effusion (MEE) and for distinguishing between MEE and AOM [6]. The diagnostic precision can be significantly improved by using tympanometry [7]. Recommendations for antibiotic treatment of AOM have changed over the last decades, and today initial observation is recommended [8]. In Denmark penicillin V is still recommended as first choice of treatment, if the GP finds it necessary to prescribe an antibiotic. Characteristics like the child's age are important to be taken into account when the GPs decide whether to prescribe antibiotics or not [8–12]. 

The aim of this study was to analyse associations between GPs' prescription of antibiotics and symptoms, diagnoses, and characteristics of children. 

## 2. Methods

### 2.1. Study Design

We conducted a cross-sectional study comprising 131 GPs and 954 children from January 2010 to June 2010. All children presenting with a new ear problem (meaning that no contacts had been made to the healthcare system regarding symptoms from ears during the previous 4 weeks) were included consecutively. Due to the GPs being busy, diseases among the GPs, and other circumstances, some of the GPs did not include all children with ear symptoms. The GPs included between 1 and 27 children each with a mean number of 7.6. During the first consultation the GPs registered symptoms, results of otoscopy and tympanometry, diagnosis, and treatment. The diagnosis of the most affected ear was used for the classification of the children. Four weeks after the first consultation, the children were examined by the GPs again in order to monitor the disease and go through the same procedure as at the first consultation. This paper presents results from the first registration only.

### 2.2. Setting

All GPs from the Region of Southern Denmark and Region Zealand (*n* = 1433) were invited to participate in the study. A total of 152 GPs or GP trainees and 45 GP nurses (who were involved in the procedure with tympanometry in the same practices) participated in a one-day course on diagnosis and treatment of otitis media. Subsequently, 131 of the GPs included children in the cohort from January 2010 to June 2010. 

### 2.3. Danish Healthcare System

Denmark has a tax-funded healthcare system, providing free access to general practices, outpatient clinics, and free hospital care for all citizens. A total of 98% of the 5.5 million Danish citizens are registered with a GP and receive free medical care. Prescribed medications are partly subsidised [13].

### 2.4. Audit Project Odense

Data for the study were collected via an audit about ear problems in preschool children carried out by Audit Project Odense (APO). APO is a quality development concept widely used in many countries [14]. It includes registration of the GPs' activities, courses, followup, and evaluation of their treatment patterns in connection with specific projects. APO data have proved to be valid for research purposes and are used in many other research projects [4, 15]. 

### 2.5. Course on Ear Symptoms, Diagnosis, and Tympanometry

The one-day course had focus on ear symptoms, diagnosis, and practical use of tympanometry in all children with otitis media symptoms. The GPs were encouraged to let their nurses participate in the course, especially designed to improve diagnosis and treatment of otitis media (OM) in general practice.

On the course an otolaryngologist taught diagnosing ear diseases and gave practical information about tympanometry. Technical details regarding the use of tympanometry were presented by representatives from Interacoustics A/S, a company producing tympanometers (http://www.interacoustics.com/). 

### 2.6. Participants

The children included were between 0 and 7 years old. Children, whose parents could not read or write Danish, were not included. Furthermore, children with continuing middle ear problems (i.e., children who had visited an otolaryngologist or their GP in connection with ear symptoms during the previous four weeks) and children with tympanostomy tubes were not included. 

### 2.7. Questionnaire

The questionnaire to the GPs was developed by the authors based on the literature reviews and clinical experience. The questionnaire was pilot-tested and minor changes were made. The questionnaire comprised questions on symptoms (fever, sleep problems, earache, ear rubbing, reduced hearing, ear fluid, delayed language development, and problems with the balance) reported by the parents. The GPs recorded results of otoscopy, results of tympanometry, the diagnosis, whether any drug was prescribed, and whether the child was referred to an otolaryngologist or a hospital. 

During the first consultation the parents were asked to bring their child to a follow-up consultation four weeks later, regardless of the fact that whether the child had ear symptoms after four weeks. 

### 2.8. Statistical Analyses

Proportions and mean values are presented with 95% confidence intervals (CIs). We used logistic regression to analyse associations between prescribing antibiotics and symptoms, medical examination, child characteristics, and GP characteristics, respectively. We accounted for possible cluster effects from children sharing the same GP by using robust cluster estimation. 

Initially, separate logistic regression analyses were made for amoxicillin (including other antibiotics) and penicillin V. Since the results of the analyses were very similar, we combined the two types of antibiotics in the further analyses to improve the statistical power. 

We tested for interactions where they seemed likely from a clinical point of view. We tested for interactions between fever and earache, fever and sleep problems, age and earache, and age and sleep problems. Due to interaction between fever and earache, the further analyses on fever were conducted separately for children with earache and children without earache, respectively. Similarly, analyses on the effect of earache were conducted separately for children with fever and children without fever. The adjusted analyses on the effects of the child's gender, sleep problems, ear rubbing, tympanometry results, otoscopy results and of GP gender and GP age included the interaction term between fever and earache. It was decided a priori to adjust for all the independent variables in the tables. 

Because of different treatment recommendations for children up to two years of age and children older than two years of age, respectively, the analyses were stratified on the two age groups, with children turning two on 1 April 2010 (date where age is estimated) in the youngest age group. STATA release 11 was used for all statistical analyses. *P* < 0.05 was considered significant.

## 3. Results

A total of 954 children were registered, 14 of whom were excluded due to the age limit. Some (55.2%) of the children were given the diagnosis of AOM, of which 71.0% (CI: 67.1–74.9) received a prescription of an antibiotic. Penicillin V was the most frequently prescribed antibiotic (21%) while amoxicillin was prescribed to 19% (Table 1). Amoxicillin was prescribed significantly less often to children older than two years of age compared to children aging two years and less (OR: 0.56, CI: 0.33–0.94). 

**Figure 1:**
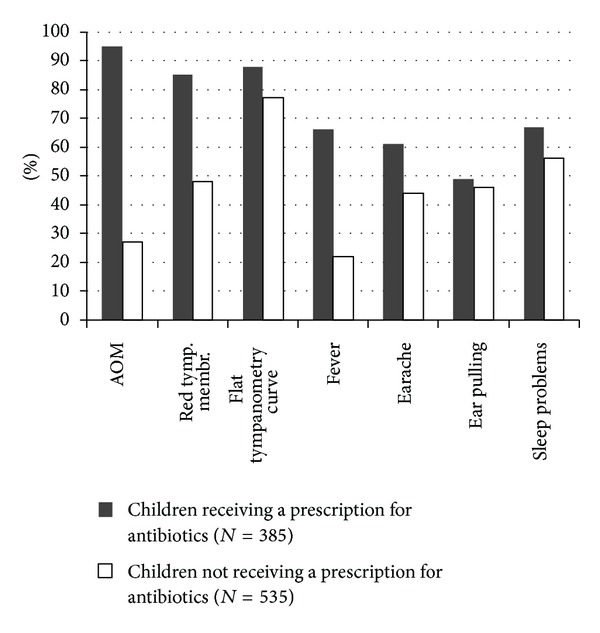
Distribution of findings, symptoms, and AOM divided into children receiving a prescription for antibiotics and not receiving a prescription for antibiotics.

Figure 1 shows that 94.5% (CI: 92.2–96.8) of children receiving a prescription of antibiotics were diagnosed with AOM, and 67.0% (CI: 62.3–71.7) of children receiving a prescription of antibiotics had sleep problems, while 66.2% (CI: 62.3–71.7) had a fever. In comparison 25.5% (CI: 22.8–30.2) of children not receiving a prescription of antibiotics were diagnosed with AOM, and 55.9% (51.7–60.1) of children not receiving a prescribing of antibiotics had sleep problems. Of the children receiving antibiotics 15 were diagnosed with MEE, most of whom had fever and were receiving antibiotics because of other infections such as tonsillitis or pneumonia (data not shown). Of all the children 375 had a fever, 254 (67.7%) of whom received a prescription of antibiotics. Among children without fever 129 (22.9%) were prescribed antibiotics, while 113 (87.6%) had a flat tympanometry curve, and 97 (75.2%) had earache. Only three children without a fever and earache and without a flat tympanometry curve received a prescription of antibiotics (data not shown).

Table 2 shows crude and adjusted ORs for the associations between prescribing antibiotics and gender, age, symptoms, and medical examination. Fever has a significant effect on children with and without earache, and it applies to both children up to two years of age and children older than two years of age. Having a flat tympanometry curve (Type B) was significantly associated with prescribing antibiotics for children older than two years. A red tympanic membrane (a result of otoscopy) was associated with receiving a prescription of antibiotics in both age groups. 

There were no significant associations between antibiotic prescribing and GP gender and GP age (results not shown). 

## 4. Discussion

### 4.1. Principal Findings

In this study we found that GPs to a large degree prescribed antibiotics appropriately according to recommendations [10, 16]. That is, fever (with or without earache) was statistically significantly associated with prescribing antibiotics for children up to two years of age and children older than two years of age. For children without fever, earache was statistically significantly associated with antibiotics in both age groups. A red tympanic membrane was statistically associated with the prescribing antibiotics for both age groups, while a flat tympanometry curve was only statistically associated with the prescribing antibiotics for the oldest children. 

### 4.2. Explanations and Comparison with Other Studies

In our study 71.0% of children with AOM received a prescription of antibiotics which is similar to another study by Hansen et al., finding that 72.0% of children with AOM received antibiotics in Denmark. Another Danish study analysing the effect of tympanometry found that GPs using tympanometry generally prescribed less antibiotics compared to a similar group not using tympanometry [7]. We expected that in this present study fewer children would receive a prescription of antibiotics as all GPs used tympanometry, which was not the case in the study by Hansen et al. The reason for the lack of differences between the two studies could be that fewer children might have been diagnosed with AOM in this present study and the ones who did receive the diagnosis might have been more ill.

It cannot be ruled out that the parents' expectations of prescribing antibiotics may have influenced the prescription of antibiotics by the GPs, as treatment practices in children with otitis media, and parental expectations earlier are found to be associated [17].

Children of two years of age or younger more often received a prescription of amoxicillin than children older than two years. A reason for this could be that it is difficult to get small children to swallow penicillin V because of the unpleasant taste. 

Most children with AOM recover spontaneously within a few days, and on average 16 children have to be treated with antibiotics to prevent one child from suffering from earache after 2–7 days [16]. It is therefore important to prescribe antibiotics to the children who are most likely to benefit from it. International guidelines recommend that, depending on clinical assessment of severity, children with bilateral acute otitis media younger than 2 years of age, acute otitis media in children with otorrhoea, or children with acute sore throat/acute pharyngitis/acute tonsillitis should be considered for immediate antibiotic treatment [8]. Apart from children with the above mentioned disorders, it is difficult to identify the children who are most likely to benefit from antibiotic treatment. 

 Prescribing antibiotics to children with earache but without fever is controversial, as the child might have acute nonsevere otitis media which is not recommended to be treated with antibiotics. Further, it is difficult to predict the developments of fever and earache in the course of an otitis media infection. Hence, delayed prescription of antibiotics may be advised for GPs when deciding whether a child with earache and no fever should receive a prescription of antibiotics [11]. 

The result of tympanometry is associated with prescribing antibiotics for the oldest children but not for the youngest children. A reason for this difference might be that most guidelines distinguish between children younger and older than two years of age. Antibiotics are often recommended for children younger than two years, while initial observation is recommended for children older than two years with mild to moderate AOM [10–12, 16]. Thus GPs might rely more on the results of tympanometry when they decide whether to prescribe antibiotics for the oldest children, while they are more inclined to prescribe antibiotics for the youngest children regardless of the results of the tympanometry. Another reason for differences between the two age groups could be that the GPs find it more difficult to get the smallest children to cooperate when performing tympanometry and consequently might regard the results of tympanometry in small children less valid than the results of tympanometry in older children. 

### 4.3. Strengths and Weaknesses

A strength of this study is that we used a data collection tool with which the GPs are familiar [18, 19]. This may have optimised the consistency in the GPs' registration of the patient data. The study was part of a quality development project for the individual practitioner; hence the GPs used data collected on their own patients for learning purposes. The GPs were encouraged to include all children with ear symptoms consecutively, and we have no reason to believe that the GPs deviated from the consecutive procedure. 

The GPs participated on a voluntary basis and may have had a particular interest in ear diseases and quality development. However, according to annual practice records of the Danish College of General Practitioners, the GP population in our study was comparable to GPs in general in Denmark with respect to age, gender, and type of practice [20].

The accuracy of the diagnoses given by the GPs might be questioned as AOM and MEE are often difficult to distinguish [21]. However, the use of tympanometry in our study is likely to have enhanced the possibility of establishing correct diagnoses, as an earlier study has shown that the use of tympanometry in combination with otoscopy has a sensitivity of 90% and a specificity of 75% for diagnosing MEE correctly [22]. Furthermore, a combination of tympanometry and otoscopy significantly reduces the number of AOM diagnoses compared to diagnoses based on otoscopy alone [7, 23], and the present study reflects enhanced clinical procedures concerning diagnosing and prescribing antibiotics. 

In theory, the order of the diagnostic and treatment processes is first diagnosis and then choice of treatment. However, we cannot rule out that diagnosis and choice of treatment interact in some way. 

By including children with all types of ear symptoms presented in general practice, we obtained a group of children where the only selection was the patients' doctor-seeking patterns. This enhances the generalisability of the study, and the results may apply in similar situations in related settings, where children present with ear symptoms in primary care. 

### 4.4. Implications for Practice and Further Research

This study demonstrated that GPs to a large degree prescribe antibiotics according to recommendations. As there is substantial variation in the course of otitis media, it is of great importance to identify factors associated with the course of otitis media, including the effects of different treatment strategies. Relevant factors could be symptoms in the beginning of the course of otitis media, socioeconomic factors, and other factors associated with the onset of otitis media. 

## 5. Conclusion

This study indicates that GPs to a large degree prescribe antibiotics according to guidelines, that is, to children with fever, AOM, a red tympanic membrane, and a flat tympanometry curve. 

## Figures and Tables

**Table 1:** 

Variable	Definition	*n*	Percent (95% CI)
Characteristics of the children		940	
			
Gender	Boys	497	52.9 (49.7–56.0)
	Girls	443	47.1 (43.9–50.3)
		940	
Age	0–≤2 years	448	47.7 (44.5–50.9)
	>2–7	492	52.3 (49.1–55.5)
		926	
Tympanometry	Other types	159	17.2 (14.7–19.6)
	Type B (flat curve)	767	82.8 (80.4–85.2)
		935	
Otoscopy	No red tym. membrane	349	36.8 (33.7–39.9)
	Red tym. membrane	591	63.2 (60.1–66.3)
		928	
	Normal	38	4.1 (2.8–5.4)
Diagnoses	OME	377	40.6 (37.5–43.8)
	MEE	513	55.3 (52.1–58.5)
		919	
	Penicillin V	201	21.4 (18.6–24.0)
	Amoxicillin	177	18.8 (16.3–21.3)
Treatment*	Other antibiotics	8	0.9 (0.3–1.4)
	Pain reliever	266	28.3 (25.4–31.2)
	Nose drops	99	10.5 (8.6–12.5)
Characteristics of the GPs		131	
			
Gender	Male	55	43.5 (34.9–52.1)
	Female	71	56.5 (47.9–65.1)
		131	
Age	27–49 years	65	48.0 (39.4–56.8)
	50–68 years	66	51.9 (43.2–60.6)

*Some children received more than one kind of treatment.

**Table 2:** Crude and adjusted odds ratio for prescribing antibiotics.

Variable	Meaning	*N*	OR (CI)	*P* values	Adjusted OR*	Adjusted *P* values
Children 0–≤2 years (*N* = 441)						

Gender	Boys	236	1		1	
	Girls	205	1.46 (1.00–2.14)	0.05	1.27 (0.82–1.97)	0.28
Symptoms						
Fever (+ earache)	No fever	68	1	<0.01	1	<0.01
	Fever (>38, 5°C)	81	5.25 (2.60–10.58)		5.89 (2.62–13.21)	
Fever (no earache)	No	162	1	<0.01	1	<0.01
	Yes	130	10.71 (6.12–18.75)		8.13 (4.61–14.32)	
Earache (+ Fever)	No	130	1	0.31	1	0.29
	Yes	81	1.37 (0.75–2.52)		1.43 (0.74–2.79)	
Earache (no fever)	No	162	1	<0.01	1	0.02
	Yes	68	2.80 (1.45–5.41)		2.32 (1.18–4.56)	
Sleep problems	No	115	1	0.25	1	0.42
	Yes	326	1.29 (0.84–1.99)		1.25 (0.73–2.13)	
Ear rubbing	No	211	1	0.20	1	0.48
	Yes	230	0.78 (0.54–1.14)		0.80 (0.44–1.47)	
Medical examination						
Tympanometry	No Type B	58	1	0.02	1	0.10
	Type B	377	2.07 (1.13–3.77)		1.92 (0.89–4.13)	
Otoscopy	No RTM	151	1	<0.01	1	<0.01
	Red tym. mem.	288	7.06 (4.33–11.50)		4.73 (2.52– 8.86)	

Children >2–7 years (*N* = 478)						

Gender	Boys	251	1	0.15	1	0.25
	Girls	227	0.76 (0.53–1.10)		0.77 (0.49–1.21)	
Symptoms						
Fever (+ earache)	No fever	210	1	<0.01	1	<0.01
	Fever (>38,5°C)	113	4.27 (2.61–6.98)		4.59 (2.55–8.25)	
Fever (no earache)	No	104	1	<0.01	1	<0.01
	Yes	49	21.88 (8.39–57.05)		19.45 (6.38–59.24)	
Earache (+ fever)	No	49	1	0.28	1	0.62
	Yes	113	1.47 (0.73–2.96)		1.26 (0.51–3.12)	
Earache (no fever)	No	104	1	<0.01	1	<0.01
	Yes	210	7.54 (3.33–17.08)		3.62 (1.50–8.75)	
Sleep problems	No	245	1	<0.01	1	0.41
	Yes	231	1.88 (1.29–2.72)		1.21 (0.76–1.93)	
Ear rubbing	No	270	1	<0.01	1	0.93
	Yes	206	1.58 (1.09–2.28)		1.02 (0.62–1.70)	
Medical examination						
Tympanometry	No Type B	97	1	0.00	1	<0.02
	Type B	373	2.12 (1.30–3.64)		2.42 (1.17–5.00)	
Otoscopy	No RTM	184	1	<0.01	1	<0.01
	Red tym. mem.	293	5.36 (3.44–8.32)		3.76 (2.13–6.64)	

*The ORs are adjusted for GPs, gender and age and for all variables included in the table.
